# Natural Variation of Heterokaryon Incompatibility Gene *het-c* in *Podospora anserina* Reveals Diversifying Selection

**DOI:** 10.1093/molbev/msu047

**Published:** 2014-01-20

**Authors:** Eric Bastiaans, Alfons J.M. Debets, Duur K. Aanen, Anne D. van Diepeningen, Sven J. Saupe, Mathieu Paoletti

**Affiliations:** ^1^Laboratory of Genetics, Wageningen University, Droevendaalsesteeg, Wageningen, The Netherlands; ^2^CBS Fungal Biology Centre, Uppsalalaan, Utrecht, The Netherlands; ^3^Institut de Biochimie et de Génétique Cellulaires, UMR 5095 CNRS, Université de Bordeaux 2, France

**Keywords:** allorecognition, fungi, innate immunity, vegetative incompatibility, somatic fusion, diversifying selection

## Abstract

In filamentous fungi, allorecognition takes the form of heterokaryon incompatibility, a cell death reaction triggered when genetically distinct hyphae fuse. Heterokaryon incompatibility is controlled by specific loci termed *het*-loci. In this article, we analyzed the natural variation in one such fungal allorecognition determinant, the *het-c* heterokaryon incompatibility locus of the filamentous ascomycete *Podospora anserina.* The *het-c* locus determines an allogenic incompatibility reaction together with two unlinked loci termed *het-d* and *het-e.* Each *het-c* allele is incompatible with a specific subset of the *het-d* and *het-e* alleles. We analyzed variability at the *het-c* locus in a population of 110 individuals, and in additional isolates from various localities. We identified a total of 11 *het-c* alleles, which define 7 distinct incompatibility specificity classes in combination with the known *het-d* and *het-e* alleles. We found that the *het-c* allorecognition gene of *P. anserina* is under diversifying selection. We find a highly unequal allele distribution of *het-c* in the population, which contrasts with the more balanced distribution of functional groups of *het-c* based on their allorecognition function. One explanation for the observed *het-c* diversity in the population is its function in allorecognition. However, alleles that are most efficient in allorecognition are rare. An alternative and not exclusive explanation for the observed diversity is that *het-c* is involved in pathogen recognition. In *Arabidopsis thaliana*, a homolog of *het-c* is a pathogen effector target, supporting this hypothesis. We hypothesize that the *het-c* diversity in *P. anserina* results from both its functions in pathogen-defense, and allorecognition.

## Introduction

Non-self-recognition is essential to many aspects of interorganismal interactions, from social behavior to defense against pathogens (Boehm and Zufall 2006; Strassmann et al. 2011). Conspecific recognition occurs between individuals belonging to the same species while heterospecific recognition occurs between individuals from different species. Genes involved in non-self-recognition often display specific evolutionary signatures such as balancing selection and fast evolution (Tiffin and Moeller 2006; Schierup and Vekemans 2008; Weedall and Conway 2010).

Filamentous fungi have the ability to undergo spontaneous somatic cell fusions. However, fusion between wild isolates usually results in a rejection response. This process is known as heterokaryon incompatibility (HI) or vegetative incompatibility. The fused hyphal compartments undergo cell death if the strains involved express incompatible alleles for one or more of the polymorphic HI genes (*het*-genes) (for review see Glass et al. [2000], Saupe [2000], and Aanen et al. [2010]).

Thus, allorecognition in the form of HI sets the boundaries for individuality, but it still is unclear why this individuality is important in the evolution of fungi (Aanen et al. 2008). One possible explanation is that allorecognition provides protection against parasitic nuclei (Hartl et al. 1975; Nauta and Hoekstra 1994; Debets and Griffiths 1998; Aanen et al. 2008; Brusini et al. 2011). Theoretical models have shown that such parasitism can select for allorecognition, as a protection against it (Nauta and Hoekstra 1996; Aanen et al. 2008, 2010). The stable outcome is a high degree of allorecognition diversity in combination with very low levels of parasitism (Czaran T, Hoekstra RF, Aanen DK, submitted for publication). Another proposed function of allorecognition is protection against the spread of cytoplasmic replicons, such as deleterious mitochondrial plasmids and mycoviruses (Debets et al. 1994; van Diepeningen et al. 1997; Brusini et al. 2011). However, HI does not always provide a strong barrier against the spread of such elements (Debets et al. 1994; Cortesi et al. 2001).

In general, each fungal species displays around ten unlinked *het*-loci, which in most cases have a limited number of alleles. This genetic constitution is sufficient to generate considerable numbers of vegetative compatibility groups (VCGs; Wu et al. 1998; Powell et al. 2007). A VCG is defined as a group of strains, which are all compatible with each other but incompatible with members of a different VCG. *Het*-genes have been characterized in the model ascomycetes *Neurospora crassa* and *Podospora anserina* (Aanen et al. 2010), and more recently in the phytopathogenic species *Cryphonectria parasitica,* causing chestnut blight (Choi et al. 2012). Many of the characterized *het*-genes encode proteins displaying a domain termed HET domain, which is involved in initiating cell death (Paoletti and Clave 2007). The level of polymorphism at *het-*loci is generally elevated (Turcq et al. 1991; Wu et al. 1998; Hall et al. 2010; Choi et al. 2012; Lafontaine and Smith 2012). In several cases, it could be shown that these loci are under an evolutionary regimen that favors generation and maintenance of polymorphism. Notably, in *N. crassa het-C* and *het-6* show positive selection, transspecies polymorphism and balancing selection, in line with their proposed role in allorecognition (Wu et al. 1998; Micali and Smith 2006; Powell et al. 2007; Hall et al. 2010).

In *P. anserina*, the *het-c, het-d* and *het-e het*-loci define two incompatibility systems (*het-c/het-e* and *het-c/het-d*). Each locus is multiallelic and each *het-c* allele is incompatible with a subset of *het-d* and *het-e* alleles. *Het-d* and *het-e* encode STAND proteins (Leipe et al. 2004) that display a N-terminal HET cell-death effector domain, a central NACHT domain for fixation of NTPs and a C-terminal WD repeat domain (Saupe et al. 1995a; Espagne et al. 2002; Chevanne et al. 2009). The WD repeat domain is a ligand-binding domain that defines specificity of interaction with the antagonistic HET-C partner (Espagne et al. 2002; Chevanne et al. 2009, 2010). *Het-d* and *het-e* are part of a large gene family termed *nwd*, comprising a total of ten members encoding proteins with a NACHT domain and the WD repeat domain. A subset of five of these genes encode proteins with an N-terminal HET domain (the *hnwd* genes). Positive selection was evidenced in the WD repeat region at the *het-d* and *het-e het*-loci (Paoletti et al. 2007). The evolution of the WD repeat encoding sequences is remarkable. The repeats of all members of the gene family undergo concerted evolution as repeat exchanges occur both within and between members of the gene family. Positive diversifying selection then operates on four specific codon positions of each 42 amino acid long WD-repeat. These four codons correspond to amino acid positions that are predicted to be located at the interaction surface of the WD repeat β-propeller structure. This concerted evolution process allows for generation of new alleles at a high frequency (Chevanne et al. 2010). Because, the *hnwd*-genes are structurally homologous to STAND proteins acting as pathogen recognition receptors (PRRs) in plants and animals, it was proposed that these genes could represent fungal PRRs (Paoletti and Saupe 2009). In that hypothesis, the observed diversity in these *het*-genes would be a consequence of diversifying selection for pathogens resistance, rather than of selection for conspecific allorecognition. The HI reaction induced by these *het*-genes would then be a by-product of their pathogen-driven diversification, and not a primary function.

*Het-c,* the interacting partner of the *het-e* and *het-d hnwd*-genes in HI encodes a glycolipid transfer protein (GLTP) (Saupe et al. 1995b). GLTPs are almost universally conserved in eukaryotes and bind and transfer various glycolipids between vesicles in vitro, but their biological function remains unclear (Mattjus 2009). Glycolipid binding and transfer activity of HET-C has been demonstrated in vitro, and the X-ray structure of the protein has been solved (Mattjus et al. 2003; Kenoth et al. 2010). In addition to its role in HI, HET-C has a function in the sexual cycle of *P. anserina* as its inactivation leads to defects in the formation of meiotic progeny (ascospores) (Saupe et al. 1994).

Originally, four *het-c* alleles (*het-c1* to *het-c4*) have been identified in a collection of 19 *P. anserina* isolates, each one being characterized by its specific incompatibility pattern with the different *het-d* and *het-e* alleles (Bernet 1967; Saupe et al. 1995b). In this article, we investigate the evolutionary features of this gene. We have screened a collection of *P. anserina* isolates of more than 100 wild isolates collected in the area around Wageningen (the Netherlands), for polymorphisms and functional diversity at the *het-c* locus. Additionally, we screened six isolates originating from various places around the world. We show that *het-c* is highly polymorphic and describe eleven different *het-c* alleles corresponding to seven functional categories in HI. We identify several codons in *het-c* that are under positive diversifying selection. Hence, *het-c* like its partner genes *het-d* and *het-e* is under positive diversifying selection. Two alternate hypotheses to account for this positive selection in *het-c* (and *het-d/het-e*) are presented. As classically proposed, the allorecognition function of these genes might drive their rapid diversification. Alternatively, these genes could have a function in host-defense and pathogen-driven divergence might as a secondary consequence lead to HI.

## Results

### Screening for Alleles

**Fig. 1.**
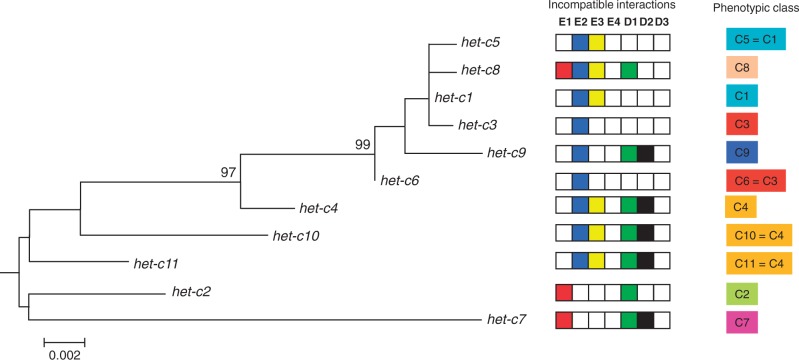
Phylogenetic relationship of the eleven *het-c* alleles identified in *Podospora anserina* in a Neighbor-Joining tree. Only bootstrap values above 95 are indicated. Branch lengths are drawn to scale according to evolutionary distances calculated as substitutions per site. In the middle panel, the incompatible interactions of each *het-c* allele with the different *het-d* and *het-e* alleles are indicated by colored boxes, white boxes indicating compatible reactions. The right panel indicates phenotypic classes as a combination of all incompatible reactions in reference to previously characterized *het-c1* to *het-c4* alleles.

**Fig. 2.**
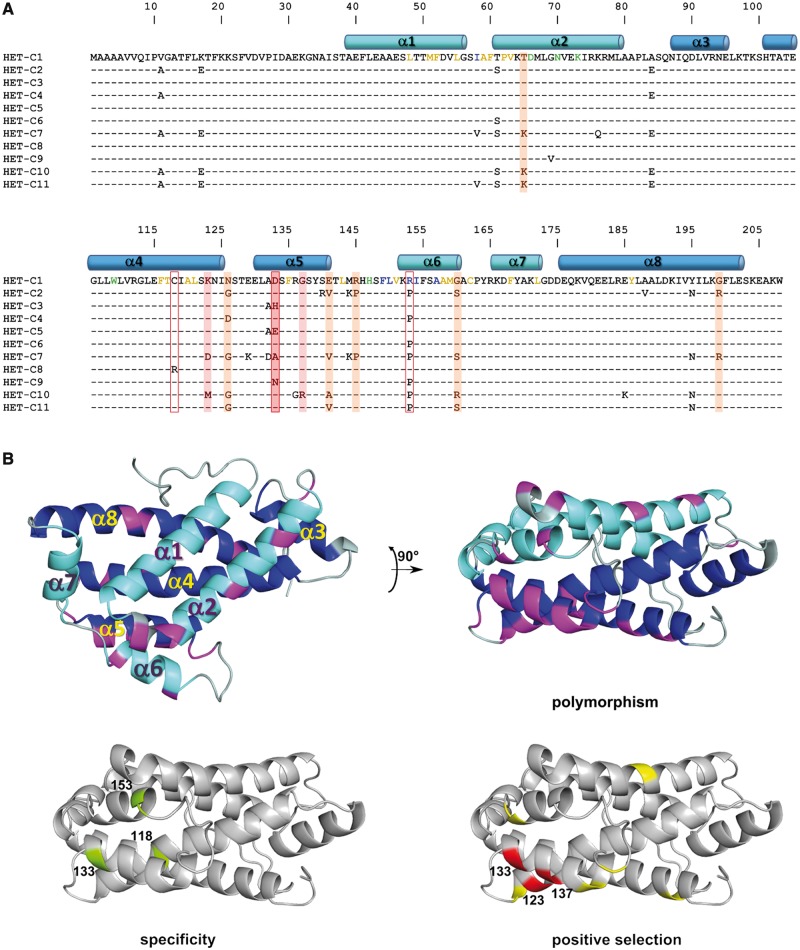
(*A*) Alignment of amino acid sequences encoded by the 11 known naturally occurring *het-c* alleles found in this study and previous studies. The amino acid sequence encoded by *het-c1* is shown completely, only sequence differences are given for the other *het-c* alleles. Position of the α-helices of HET-C is given in cyan and blue, respectively, for the helices forming the two layers of the sandwich motif. Residues colored in yellow correspond to residues involved in lipid binding, in green to residues involved in sugar binding, and in blue to residues proposed to interact with membranes. Residues boxed in red (118, 133, 153) are shown to be involved in allele specificity. Residues shadowed in red and orange correspond to residues under positive selection with a 99% and 95% confidence level, respectively. (*B*) Three-dimensional structure of HET-C protein (Kenoth et al. 2010). The α-helices that form the two layers of the sandwich motif (α1, α2, α6, α7 and α3, α4, α5, α8) are given in cyan and blue, respectively. On the two top panels, polymorphic positions are given in magenta. The two lower panels identify the residues shown to be involved in allele specificity in green and the residues under positive selection in red for residues with a 99% confidence level and in yellow for residues with a 95% confidence level.

The *het-c* gene was polymerase chain reaction (PCR) amplified and sequenced from isolates of the Wageningen *P. anserina* collection (van Diepeningen et al. 2008), along with six additional isolates from elsewhere. Among 110 isolates from the local population surrounding Wageningen, we found eight different *het-c* alleles. Five of these alleles (*het-c5* to *het-c9*) were new and not previously described, whereas the other three were identical to the ones found previously in French isolates (Saupe et al. 1995b). Additionally, six strains isolated from various places around the world yielded two additional *het-c* alleles (*het-c10* and *het-c11*). With the seven new alleles found in this study, the total number of known *het-c* alleles is now 11. A phylogenetic reconstruction of these sequences is presented in figure 1. Together, these alleles contain 50 polymorphic sites. Nine polymorphic sites are located in one of the two introns. Among the 41 remaining polymorphic sites, 6 polymorphisms are silent (discussed later and supplementary fig. S1, Supplementary Material online). The remaining differences lead to 26 polymorphic sites at the protein level (fig. 2*A*). We found no indel polymorphism in the *het-c* sequences*.*

### Mapping of the Polymorphic Sites on the HET-C Structure

*Het-c* encodes a GLTP and the structure of the HET-C protein has been solved by Kenoth et al. (2010). HET-C displays a distinctive two-layer “sandwich-motif” dominated by α-helices. Alpha-helices α1, α2, α6, and α7 make up one layer while α3, α4, α5, and α8 make up the other layer. Amino acids forming the hydrophobic pocket accommodating the lipid or amino acids involved in binding the sugar moiety upon loading of the glycolipid have been identified (fig. 2). In addition, a computational approach was used to predict amino acids potentially involved in the binding of HET-C to membranes.

An alignment of the 11 HET-C protein sequences presented in figure 2*A* reveals that the polymorphic positions are mainly clustered in two highly variable regions between positions 58 and 84 (six polymorphic sites) and positions 118 to 160 (12 polymorphic sites) thereby confirming previous observations by Saupe et al. (1995b). Eighteen polymorphic positions are located in the α1, α2, α6, α7 layer of the protein, and 12 in the α3, α4, α5, α8 layer. Six of the remaining polymorphic sites are located in loop regions between α-helices. The polymorphic positions identified between the *het-c* alleles are distinct from the positions directly involved in lipid or sugar binding consistent with the necessity to maintain *het-c*-function in glycolipid transfer (fig. 2*A*). A number of polymorphic sites are immediately adjacent to residues involved in sugar binding. Polymorphic positions are located at the protein surface and importantly, they are found in the two distinct layers of the “sandwich-motif” and thus do not group as a single interaction surface (fig. 2*B*). The existence of these two distinct polymorphic interaction surfaces might be explained in a model postulating that the WD-repeat domain of HET-D and HET-E are organized as two distinct β-propellers forming a clamp-like structure around HET-C as occurs in the cytochrome *c*/APAF-1 interaction (Yu et al. 2005).

### Specificity of the Novel *het-c* Alleles

To assess HI specificity of the newly identified *het-c* alleles, the alleles were PCR amplified along with promoter and terminator sequences, cloned and introduced by transformation in a *Δhet-c het-e4 het-d3* recipient strain. The *het-e4 het-d3* genotype was chosen for the recipient because these *het-e* and *het-d* alleles were found to be universally compatible with all previously identified *het-c* alleles. Each transformation resulted in a comparable number of transformants, suggesting compatibility between the transformed *het-c* alleles and genes expressed by the recipient strains, in particular the *nwd*-gene family members. Transformants were selected and presence of the transforming DNA was confirmed by PCR for at least two transformants for each *het-c* allele.

The seven new *het-c* alleles were tested for incompatibility with the seven known tester alleles for *het-d* and *het-e*. The results were compared with the patterns of the four original *het-c* alleles (fig. 1) (Bernet 1967; Saupe et al. 1994). Each of the 11 alleles is incompatible with at least one of the three *het-e* tester strains. As expected none of the alleles showed incompatibility to the *het-d3* or *het-e4*. The 11 *het-c* natural alleles can be grouped into 7 different phenotypic classes based on their patterns of HI, the new variant alleles defining 3 new phenotypic classes (fig. 1). The novel *het-c5* and *het-c6* alleles have the same interaction specificity as previously characterized for *het-c1* and *het-c3,* respectively. Similarly, *het-c10* and *het-c11* have the same specificity as *het-c4*. This analysis thus identifies a total of seven functional categories in wild-type alleles. In a previous study, 10 artificial chimeric alleles yielding novel interaction patterns were generated by combining sequences from the 4 reference alleles (Saupe et al. 1995b), their interaction patterns are recalled in (supplementary fig. S2, Supplementary Material online). The pattern of the novel *het-c7* allele was obtained for three of the artificial chimeric alleles. Two additional patterns are only seen in the chimeric artificial alleles.

Inspection of the compatibility of *het-c* alleles reveals existence of specific patterns in these interactions. First of note is the fact that all wild-type alleles produce at least one incompatibility reaction. In contrast to the *het-d* and *het-*e loci, no neutral alleles are found. All alleles are incompatible at least with either *het-e1* or *het-e2*. Incompatibilities with *het-e1* and *het-e2* are generally mutually exclusive. *Het-c8* represents an exception in that regard. Then, the incompatibility patterns reveal a hierarchy in the incompatibility reactions. For instance, wild-type alleles that are incompatible with *het-e1* are also incompatible with *het-d1* (only artificial *het-c2-2* is an exception)*.* Similarly, alleles incompatible with *het-d2* are also incompatible with *het-d1* and alleles incompatible with *het-e3* are also incompatible with *het-e2.* In each case, the converse is not true, that is, alleles incompatible with *het-e1* are not necessarily incompatible with *het-d1.*

The new alleles contribute to refinement of our understanding of the role of polymorphic positions in allele specificity. By comparing the four initial alleles as well as chimeric alleles, Saupe et al. (1995b) could identify that polymorphic position 133 and 153 are involved in allele specificity. This study now identified position 118 as also involved in the control of specificity. *Het-c1* and *het-c8* have a distinct incompatibility pattern and differ by a single amino acid at position 118. Replacement of the cysteine at this position in *het-c1,* by a positively charged arginine in *het-c8,* leads to the acquisition of two additional HI reactions with *het-e1* and *het-d1*. The present data also confirm the role of the amino acid at position 133. Indeed, alleles *het-c1*, *het-c3,* and *het-c5* differ only by a single polymorphism at this position. The products of *het-c1* and *het-c5* differ by a conservative substitution (of aspartic to glutamic acid) at that position and present the same incompatibility specificity (incompatible with *het-e2* and *het-e3*). A histidine is found in the *het-c3* product and this allele product is incompatible only with *het-e2*. Thus, a histidine at amino acid position 133 in place of a negatively charged amino acid determines incompatibility with *het-e3*. This brings the number of positions identified as involved in defining incompatibility specificity to three (118, 133, and 153). However, previous studies have shown that at least one polymorphic position in the N-terminal part of the protein between position 11 and 65 is also involved. The smallest combination of amino acid positions that could account for all differences in allele specificity is the seven amino acids at positions 17, 65, 84, 118, 126, 133, and 153.

Of note is the fact that allele products with different protein sequences can display the same specificity. For instance, *het-c4* and *het-c10* display the same allelic specificity while differing by ten amino acids. Similarly, *het-c7* and *het-c1-2* (a chimeric allele) are identical in specificity while differing also by ten residues. This observation may indicate that some of the polymorphisms existing in *het-c* do not modify allele specificity or that these are specific to yet unknown alleles of *het-d* and *het-e*.

### Escape from Self-Incompatibility Caused by Newly Identified *het-c* Alleles Occurs by Repeat Loss in the Antagonistic *hnwd* Gene

Self-incompatible strains caused by *hnwd*-genes escape HI most of the time through deletion of a variable number of WD repeats (Chevanne et al. 2010). A *het-d2* tester strain was crossed with a *Δhet-c* strain transformed with either *het-c9* or *het-c10*. Self-incompatible progeny were recovered and escape mutants were selected as previously described (Chevanne et al. 2010) and the length of the WD repeat domain of *het-d* analyzed by PCR. As expected most of them displayed a WD repeat domain reduced in size compared with the starting allele (data not shown). Out of the 32 escaped mutants tested, 26 were reduced in size. Their estimated size varies between 2 and 13 WD repeats, whereas the parental *het-d2* was estimated to have 12 WD repeats. Thus, escape from the genetic conflict generated by introduction of a new *het-c* variant is resolved through the modification of the WD-repeat domain of the antagonistic *het-d* gene, providing further evidence that incompatibility indeed occurs between the variant *het-c* allele and the *hnwd* locus.

### Distribution of *het-c* Alleles in the Population

**Fig. 3.**
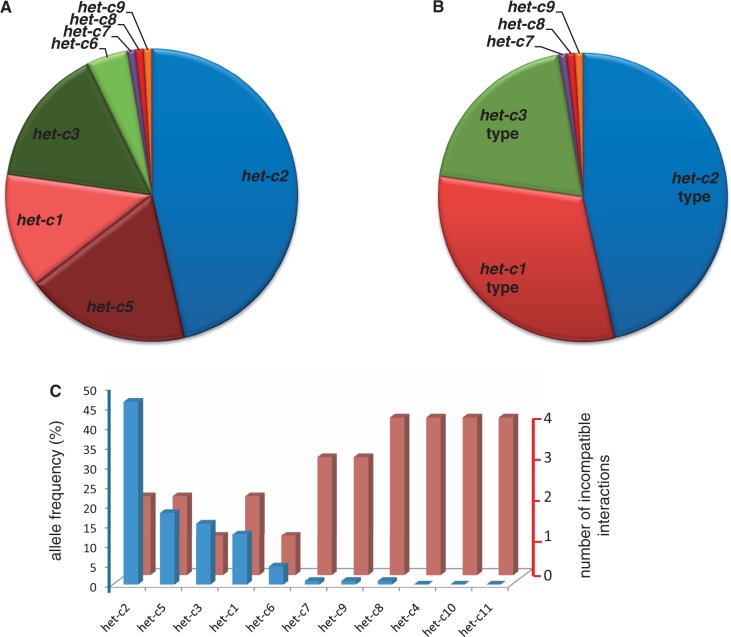
Allele frequency distribution for *het-*c in the Wageningen collection of *Podospora* anserina. (*A*) Pie chart showing frequency distribution of the eight different alleles found in the collection. (*B*) Pie chart grouping the different *het-c* alleles into functional groups based on their incompatibility pattern with known *het-d* and *het-e* alleles. (*C*) Column chart comparing for all known *het-c* alleles its allele frequencies in the Wageningen collection with the number of *het-d* and *het-e* alleles it shows an incompatible interaction with.

*Het-c2* is the most frequent allele representing 46% of the population (fig. 3*A*). Allele frequencies of *het-c1, −3,* and −*5* are in the range of 15% followed by *het-c6* at a frequency of 5%. *het-c7, −8,* and −*9* are rare alleles each found only once, while *het-c4*, −*10,* and −*11* were absent from the Wageningen population sample. Alleles can be grouped by specificity-type into phenotypic classes. *Het-c5* belongs to the same phenotypic class as *het-c1* and likewise *het-c6* is phenotypically identical to *het-c3*. This classification into phenotypic classes reveals three abundant classes (C2, C1, and C3 type) representing, respectively, 46, 31, and 20% of the population (fig. 3*B*). We compared this phenotypic class distribution with the distribution found in a collection of 19 isolates collected in various locations in France in the 1940s (supplementary fig. S3, Supplementary Material online). In this collection also, the C2-type is the most abundant type followed by C1 and C3. The C4-type allele absent from the Wageningen population was found only twice in the French isolates.

As noted previously, the vast majority of the alleles are incompatible with either *het-e1* or *het-e2*. By grouping alleles by this criterion, it appears that the alleles from the Wageningen population form two equilibrated classes with 47% and 52% of the alleles incompatible with *het-e1* and *het-e2,* respectively (the remaining 1% is incompatible with both *het-e1* and *het-e2*). Similarly, in the French collection of 19 strains, one finds 9 alleles incompatible with *het-e1* and 10 alleles incompatible with *het-e2*.

A phylogenetic reconstruction of the different *het-c* DNA sequences reveals two main clades (fig. 1). Although these clades are not well supported, it appears that alleles incompatible with *het-e2* comprise one clade, whereas two out of three alleles incompatible with *het-e1* comprise the other clade (fig. 1). Assuming that this phylogeny represents the historical relationship among these alleles, these data may suggest that these incompatibilities are ancestral and were maintained throughout evolution. Only *het-c8* seems to have gained incompatibility with *het-e2* secondarily. This situation seems to be different for incompatibility with *het-d1*, *het-d2,* and *het-e3* that seem to have been gained or lost independently several times. However, the observed clustering of these different kinds of incompatibility interactions in the phylogeny may also be a direct consequence of sequence similarity among alleles with similar incompatibility interactions caused by positive selection for their function in these incompatibility interactions.

To find a possible cause for the unequal distribution of the *het-c* alleles and the absence of *het-c4, het-c10,* and *het-c11*, we tried to find out if there is a relation with the number of incompatible reactions a *het-c* allele can cause. The *het-c* alleles differ by the number of incompatibility reactions with *het-d* and *het-e* alleles they produce. This number ranges from 1 (e.g., for *het-c3*) to 4 (e.g., for *het-c8*). There appears to be an inverse correlation between the number of incompatible interactions and allele frequency (fig. 3*C*). Interestingly, alleles that yield many incompatible reactions with the known *het-d* and *het-e* alleles are rare or absent from the population, suggesting the allorecognition function of *het-c* comes at a cost.

Natural populations of *P. anserina* are infected by a mitochondrial senescence plasmid pAL2-1, which affects life span in all tested conditions. Propagation of this deleterious plasmid was found to be limited by incompatibility based on a distinct allelic *het*-system (the *het-s/het-S* allele incompatibility system; Debets et al. 2012). We have analyzed the distribution of the senescence plasmid in relation with the *het-c* genotype (supplementary fig. S4, Supplementary Material online). It appears that the *het-c2* class of isolates that occurs at the highest frequency has a higher frequency of pAL2-1 infections compared with the less frequent *het-c* classes. However, overall we found no significant correlation between the *het-c* genotype and the distribution of the pAL2-1 plasmid.

### *Het-c* Is Subject to Positive Selection in *P. anserina*

Sequence analysis of the first four alleles unraveled unusual evolutionary features for *het-c*: More polymorphisms were identified within the open reading frame (ORF) than in the introns or 5′ and 3′ noncoding sequences (Saupe et al. 1995b). In this study, we focused our analysis on the coding sequences. We found a total of 41 polymorphic sites between the *het-c* alleles in the ORF, only six of which correspond to synonymous substitutions. Four of these synonymous substitutions occur in *het-c7* (supplementary data S1, Supplementary Material online). These observations suggest that the *het-c* locus may be under positive diversifying selection. We have thus analyzed *het-c* divergence using the HyPhy software package. The codon-based substitution models developed by Nielsen and Yang (1998) and Yang et al. (2000) were implemented within the HyPhy software package (Kosakovsky Pond and Muse 2005). These models, based on the ratios between nonsynonymous and synonymous substitutions *ω* = d*N*/d*S*, allow for varying rates of substitutions between sites to account for varying selection forces. The analysis was conducted with the *het-c* allele data set. All models except Model 4 resulted in overall d*N*/d*S* ratio values between 1.12 and 1.48 (table 1). Models 1 and 7 do not allow for *ω* >1. These results indicate that *het-c* evolves under positive selection in *P. anserina*. Comparing pairs of models (M1–M2) and (M7–M8) indicate that both models M2 and M8 identify codons under positive selection at the 99% confidence level. Posterior probabilities of model M2 reveal codons 123, 133, and 137 as positively selected at the 99% confidence level and codons 65, 126, 141, 145, 160, and 199 at the 95% confidence level. Model M8 identifies all of these codons as positively selected at the 99% confidence level, and additional codons 11, 17, 58, 69, 76, 84, 118, 129, 132, 140, 153, 185, 188, and 195 as positively selected at the 95% confidence level. Other codons are under purifying selection. This indicates that the main force driving evolution of *het-c* alleles is positive diversifying selection acting on a limited number of codons. The positions under selection are indicated on the structure of the HET-C protein (fig. 2). Positively selected positions are located on the two opposite sides of the “sandwich-motif.” The three positions that show the strongest statistical support for positive selection (123, 133, and 137) group on the HET-C structure (fig. 2*B*). Among them, position 133 is proven experimentally to be involved in allele specificity.

**Table 1.** Likelihood Values and Overall d*N*/d*S* Ratio Obtained for the Nielsen Yang Models with the 11 *het-c* Alleles from *Podospora anserina*.

Model	Log Likelihood	d*N*/d*S*
M0	−1,183.50160	1.24
M1	−1,179.03409	0.44
M2	−1,168.45456	1.42
M3	−1,168.43569	1.32
M4	−1,170.53317	0.74
M5	−1,169.07790	1.41
M6	−1,169.06485	1.45
M7	−1,179.73459	0.49
M8	−1,168.64954	1.12
M9	−1,169.06542	1.40
M10	−1,169.06280	1.42
M11	−1,169.07282	1.48
M12	−1,168.47380	1.42
M13	−1,169.06289	1.43

We next asked whether positive selection is a general feature of GLTP encoding genes in fungi. Sequences from different fungal GLTPs were recovered after blastx searches of the fungal genome sequences in the NCBI Fungal Genome Central database using the *P. anserina het-c2* sequence as a query. The accession numbers for 37 sequences retrieved are listed in supplementary figure S5 (Supplementary Material online). The chosen sequences cover the subphylum of the Pezizomycotina. A protein-guided alignment of the ORF sequences was generated with ClustalW, and a Neighbor-joining tree constructed using the MEGA4 package (Tamura et al. 2007; supplementary fig. S5, Supplementary Material online). All models found overall low d*N*/d*S* ratios of 0.15 to 0.34 (table 2). We thus conclude from this analysis that GLTP encoding genes in filamentous fungi are evolving under purifying selection and that positive selection appears specific to *P. anserina*. This implies that in *P. anserina* the GLTP *het-c* has gained a function in non-self-recognition in addition to its function as a GLTP.

**Table 2.** Likelihood Values and Overall d*N*/d*S* Ratio Obtained for the Nielsen Yang Models with the GLTP Encoding Genes from Filamentous Fungi.

Model	Log Likelihood	d*N*/d*S*
M0	−12,323.53746	0.15
M1	−12,153.94895	0.29
M2	−12,153.74433	1.70
M3	−11,960.78952	0.18
M4	−12,193.55755	0.34
M5	−11,961.46353	0.18
M6	−11,961.46354	0.18
M7	−11,959.66209	0.19
M8	−11,953.08587	0.19
M9	−11,959.35832	0.19
M10	−11,959.66246	0.19
M11	−11,960.89653	0.19
M12	−11,958.65961	0.19
M13	−12,006.13631	0.50

It is relevant to note that Ellison et al. (2011) conducted an analysis of population genomics and local adaptation in *N. crassa*, a close relative to *P. anserina*. This analysis examined single nucleotide polymorphisms (SNPs) in the transcriptome of 48 wild isolates from the Caribbean basin, South America, and Africa, constituting two recently diverged populations and a number of outliers. They identified a list of 12 genes evolving under positive selection, which did not include NCU07947, the *N. crassa* ortholog of *het-c*. In the SNPs data, we identified 18 polymorphic positions in NCU07947, all corresponding to synonymous sites (supplementary fig. S6, Supplementary Material online). Thus, as for fungal GLTP encoding genes, it seems that at least in the populations analyzed NCU07947 evolves under purifying selection. A similar genomic population analysis was conducted with two *Coccidioides* species (Ellison et al. 2011) and again we found that *Coccidioides het-c* ortholog appears to be evolving under purifying selection (supplementary fig. S7, Supplementary Material online). For a number of additional species, *het-c* allele sequences are held in databases that again display essentially synonymous polymorphisms (supplementary fig. S8, Supplementary Material online). 

Overall, it appears that fungal GLTPs encoding genes are under purifying selection while diversifying selection is operating specifically on *P. anserina het-c.*

## Discussion

We show that *het-c*, involved in vegetative incompatibility with *het-d* and *het-e* and encoding a GLTP, contains several codons that are under positive diversifying selection leading to high levels of polymorphism. In addition, we show that the fast evolving codons of *het-c* encode amino acids known not to be involved in glycolipid binding nor in forming the proposed membrane interface. Polymorphic residues are found in two regions lying at the surface on opposite sides of the protein structure. Positive selection appears to be specific to the *P. anserina het-c* gene as *het-c* orthologs in other fungal species evolve under purifying selection. We observed a high allelic diversity of *het-c* associated with an uneven allele distribution. However, the distribution of phenotypic classes, as defined by their incompatibility pattern with *het-d* and *het-e*, proves to be more balanced. The high level of diversity and polymorphism observed at the *het-c* locus was also observed at the antagonistic loci *het-d* and *het-e* (Paoletti et al. 2007; Chevanne et al. 2010), suggesting that *het-c, het-d,* and *het-e* are under similar selective regimens.

### Allorecognition Function of *het-c* as a Cause for Diversifying Selection

What is the cause of the diversifying selection acting on *het-c*? The simplest and most obvious hypothesis is that diversifying selection acting on *het-c* relates to the role of this gene in allorecognition. Classically, it is proposed that allorecognition in fungi in the form of vegetative incompatibility may have evolved to provide individuals with a protection against nuclear parasites (Hartl et al. 1975; Nauta and Hoekstra 1994; Debets and Griffiths 1998; Aanen et al. 2008; Brusini et al. 2011) or deleterious cytoplasmic elements (Debets et al. 1994; van Diepeningen et al. 1997; Brusini et al. 2011). HI will limit cytoplasmic exchanges between individuals and thus limit propagation of deleterious cytoplasmic elements between strains. The stable outcome of HI evolution is a high degree of allorecognition diversity in combination with low levels of parasitism (Brasier 1988; Muirhead et al. 2002; Czaran T, Hoekstra RF, Aanen DK, submitted for publication). In these systems, diversity is maintained by balancing selection leading to an even distribution of incompatible alleles. A typical example of this situation is provided by the *het-c* and *het-6* incompatibility systems in the genus *Neurospora*, where highly polymorphic incompatible alleles are maintained in the population at these loci over long periods of time (Wu et al. 1998; Powell et al. 2007).

Natural populations of *P. anserina* are infected by a mitochondrial senescence plasmid pAL2-1, which affects life span in all tested conditions. Plasmid infection was found to be negatively correlated with allele frequency at the *het-s* locus meaning that strains bearing the less common *het-S* allele were less infected than those bearing the more frequent *het-s* allele (Debets et al. 2012). We found no clear evidence for biased distribution of pAL2-1 in strains bearing rare alleles of *het-c* yet*.* As we are dealing here with a nonallelic system, efficiency of a *het-c* allele in preventing plasmid spreading in the population is not expected to be determined solely by its prevalence but also by the prevalence of the antagonistic *het-d* and *het-e* alleles (which is currently unknown for the Wageningen population).

### Pathogen-Driven Divergence as a Possible Alternative Cause for Diversifying Selection in *het-c*

An alternative hypothesis to explain *het-c* diversification stems from the studies on *acd11*, the *het-c* homolog in *A. thaliana*. *Acd11* (accelerated cell death) was isolated as a gene whose inactivation initiates cell death in the form of a hypersensitive response (the pleiotropic inflammatory response associated with the immune response to viral or bacterial pathogens in higher plants). The *acd11* point mutant alleles affected in glycolipids binding suppress the *accelerated cell death* phenotype indicating that the cell death reaction cannot be directly attributed to a defect in glycolipid transfer activity (Petersen et al. 2008). Instead, this cell death reaction was equated to an autoimmune reaction and is currently interpreted in the frame of the “guard hypothesis.” In this model, plant immune NB-LRR receptor proteins act as guards that survey a guardee, that is a host protein targeted by a pathogen effector molecule (Dangl and Jones 2001; Jones and Dangl 2006). The hypersensitive immune response is initiated when the receptor senses modification of the guardee by a pathogen effector molecule. It was found that the presence of the protein *NB-LRR* resistance gene *LAZ5* is required to initiate the *acd11* associated PCD reaction (Palma et al. 2010). This led to the suggestion that ACD11 might be a guardee targeted by a pathogen effector molecule. In support of this model is the fact that several proteins interacting with ACD11 were identified as direct targets of pathogen effectors (Dreze et al. 2011; Mukhtar et al. 2011). By analogy, *P. anserina,* HET-C might correspond to a guardee (pathogen effector target) under the surveillance of the *het-e* and *het-d* HNWD proteins, which are structurally related to plant NB-LRR and animal NOD-like PRRs. Plant host targets have been found to show diversifying selection consistent with the existence of a host–pathogen arms race (Kaschani et al. 2010). If *het-c* also represents a host target as proposed for ACD11, it may be speculated that diversifying selection in *het-c* is pathogen driven. The fact that pathogen-driven divergence can lead to genetic conflicts is illustrated by the process of hybrid necrosis in plants. In *A. thaliana*, hybrid necrosis occurs in the progeny of crosses between incompatible isolates. This necrosis, caused by incompatible gene-to-gene interactions, involves a pathogen resistance gene of the NB-LRR type (Bomblies et al. 2007) and phenotypically mimics an inflammatory response. Hybrid necrosis is thus considered an auto-immune condition caused by divergence in genes with an immune function.

If *het-c* indeed is a pathogen-effector target, diversification of *het-c* might result from selection of variants insensitive to the pathogen effector. Hörger et al. (2012) have recently demonstrated that co-evolution between the guardee and its guard was the main force driving the evolution of the *RCR3* gene in the wild tomato species *Solanum peruvianum*. If HET-D and HET-E act as guards of HET-C, *het-c* could also diversify rapidly to match the fast evolution of these *hnwd* genes to maintain an optimum guard–guardee interaction.

*Podospora anserina* is likely to encounter many potential pathogens and parasites during its life. It grows on herbivore dung where many other organisms occur like bacteria, fungi, insects, and nematodes. To grow and survive in such a hostile environment a good defense mechanism against all the potential enemies, it encounters in the dung would seem beneficial. Although currently no pathogens of *P. anserina* are known, a number of fungal species have been described as interfering with *P. anserina*’s development (Silar 2005). Mycoviruses have long been described in the fungal kingdom (Dawe and Nuss 2012), and it is also not uncommon for bacteria to be pathogenic to fungi (Paoletti and Saupe 2009; Frey-Klett et al. 2011). Of note in this context, is the fact that the transcriptional profile during the incompatibility response in *Podospora* is reminiscent of a defense response with induction of numerous toxins, secondary metabolism clusters and hydrolases (Bidard et al. 2013).

### *Het-c* Allele Distribution and Polymorphism

It is of note that all *het-c* identified in this study are active in incompatibility although *het-c* alleles inactive in VI but retaining *het-c*-function in ascospore formation can be obtained artificially (Saupe et al. 1995b). If *het-c* HI is the nonadaptive accidental by-product of pathogen-driven divergence, one does not expect that variant alleles systematically trigger incompatibility. The fact that all *het-c* alleles show HI reactivity rather favors the notion that HI is indeed adaptive and that alleles are selected for that purpose. In addition, although *het-c* allele distribution is unbalanced, when considering *het-c* allele reactivity to *het-e1* and *het-e2*, functional incompatibility classes reach close to balanced distribution in populations and thus match expectations for an allorecognition system under balancing selection.

Although all identified *het-c* alleles display activity in incompatibility against at least one *het-d* or *het-e* allele, the opposite is not true. Indeed, null *het-d* and *het-e* alleles for incompatibility exist (*het-e4* and *het-d3*). Bernet (1967) showed that these inactive alleles were widely represented in the 16 isolates from France: 6 isolates expressed the inactive *het-e4* allele, and 13 expressed the null *het-d3* allele (including five also expressing the *het-e4* null allele). An important consequence of the presence of null *het-d* and *het-e* alleles is that these incompatibility systems fail to split the population into so-called VCGs. Members of a VCG are compatible with each other and incompatible with members of other VCGs. In principle, individuals belonging to a given VCG will be protected from the infectious replicons present in isolates from different VCGs. Infection may spread even between incompatible strains when passing through an intermediate strain showing dual compatibility. The existence of such inactive alleles (at least in the Bernet collection) questions the notion that *het-c/het-d* and *het-c/het-e* incompatibility systems are adaptive. Yet, it might be that existence of such null alleles is an unavoidable consequence of the genetic instability of the WD-repeat regions of the *hnwd* genes (Chevanne et al. 2010).

Most isolates in the Wageningen population express the *het-c* alleles that are less efficient in allorecognition (*het-c1*, *het-c2*, *het-c3*, *het-c5*, and *het-c6*), these alleles only display either one or two incompatible interactions with the known *het-d* and *het-e* testers. In contrast, the most efficient *het-c* alleles in terms of allorecognition (*het-c4*, *het-c7*, *het-c8*, *het-c9*, *het-c10,* and *het-c11*), incompatible with three or four tester strains, are either absent from the Wageningen population or found in only one isolate. Again, this is not expected if the allorecognition function is considered adaptive. The rare occurrence of *het-c* alleles leading to numerous incompatible interactions could be explained by the cost of the genetic conflicts such alleles trigger during the sexual cycle. Non-allelic systems in *P. anserina* also act as sexual incompatibility loci. Crosses between incompatible strains lead to partial or total sterility and the formation of self-incompatible lethal progeny (Bernet 1967). In an event of outcrossing, strains carrying *het-c* alleles leading to multiple incompatibilities (i.e., *het-c8*) might be disfavored because they are unlikely to encounter a compatible sexual partner.

If polymorphism in *het-c* is solely driven by the allorecognition function, it might be expected that all variable positions impact allele specificity. Position 133 illustrates this principle. This position is the most variable in HET-C (with five distinct amino acids found in the different allele products), and it was indeed found to control allele specificity. Yet, part of the polymorphism in *het-c* is not accounted for in terms of functional variation in the incompatibility specificity. That is a number of polymorphisms do not lead to changes in the incompatibility pattern with the currently known *het-d* and *het-e* alleles. This is for instance illustrated by the fact that the *het-c4* and *het-c10* encoded proteins display the same allelic specificity while differing by ten amino acids. Simply put, there is apparently more polymorphism in *het-c* than needed for the allorecognition function. Thus, positive selection detected on these codon positions may not be caused by the HI function. One possibility is that these polymorphisms are the result of selection for pathogen defense. Another possibility is that the additional polymorphisms are compensatory mutations to maintain its GLTP function. Alternatively, we might have an incomplete picture of the incompatible interactions. Additional *het-d* and *het-e* alleles with different specificity types might exist in the Wageningen population. This last hypothesis appears quite likely considering that many more *het-c* alleles were identified in this study in addition to the original alleles identified in the Bernet collection that comprised only 16 strains. Finally, as mentioned before during outcrossing, self-incompatible progeny can arise. There will then be high selective pressure to resolve this conflict, so any mutations that can remove the self-incompatibility will be selected, and this could potentially lead to new polymorphisms. As we observed, resolution of such conflicts are more likely to generate polymorphisms in the WD repeat domains of *het-d* or *het-e* but will also on occasion produce mutations in *het-c*. This may also explain why we see some alleles at a very low frequency. They may have been selected upon an outcrossing event to escape self-incompatibility, while there is no subsequent selective benefit that would increase their frequency.

Clearly, the cause for rapid variation of *het-c* cannot at present be unambiguously defined. If selection for allorecognition function is apparently a satisfying proximal cause for this diversification, certain aspects in *het-c* allele distribution and polymorphism are not fully accounted for in this hypothesis. A more complete understanding of the evolution of *het-c* may have to await a full characterization of the allele constitution and distribution at the partner *het-d* and *het-e* alleles in the Wageningen population.

### Incompatibility as an Exaptation

The term exaptation was introduced to describe the reuse by natural selection of a structure or gene with a previously different purpose (Gould and Vrba 1982). Mating-type incompatibility in *N. crassa* may constitute an example of this form of exaptation: unlike in most Pezizomycetes, in *N. crassa* the mating-type locus has a secondary function as a HI locus. The *het-s*-based incompatibility in *P. anserina* was also proposed to result from an exaptation event (Debets et al. 2012). Actually, considering that all HI systems characterized so far include at least a gene conserved across the fungal kingdom and not involved in HI in other species (Saupe et al. 2000; Kerényi et al. 2006; Micali and Smith 2006; van Diepeningen et al. 2009; Iotti et al. 2012), lineage-specific exaptation of a conserved cellular function to ensure allorecognition function might be the rule rather than the exception. If members of the *het-c* and *het-d/e* are indeed involved in pathogen recognition in a guardee/guard relationship, it is possible that incompatibility between *het-e* (and *het-d*) and *het-c* variants has emerged as a by-product of this pathogen-driven diversification. As HI can be selectively advantageous in a number of conditions, from then on, selection might have acted on *het-c*/*het-e* to maintain and promote polymorphisms leading to incompatibility. According to this last hypothesis, *het-c* would be under a dual selection regimen and diversification may represent the additive result of pathogen-driven divergence and selection associated with the allorecognition function.

## Materials and Methods

### Strains and Culture Conditions

*Podospora anserina* strains used for *het-c* screening are from the Wageningen collection of wild-type isolates and from six strains collected worldwide which were retrieved from the CBS, Utrecht, The Netherlands (www.cbs.knaw.nl, last accessed February 3, 2014). The strain numbers for the isolates retrieved from the CBS are as follows: CBS124.78, CBS253.71, CBS333.63, CBS433.50, CBS455.64, and CBS102042. In total, 110 isolates were used from the Wageningen collection of wild-type *P. anserina* strains, that were all collected at different locations near Wageningen between 1991 and 2001. Most strains have been isolated from rabbit, horse, or sheep dung. Details on the sampling methods and locations of the Wageningen collection have been described earlier (van der Gaag et al. 1998; van Diepeningen et al. 2008).

### DNA Isolation, PCR, and Sequencing

DNA was isolated from these strains by growing them in a Petri dish with *P. anserina* synthetic medium (PASM, described by Esser [1974]) covered with a layer of cellophane foil. After 2 days of growth at 27 °C, mycelium was scraped off and put in a 1.5 ml Eppendorf tube, which was then frozen in liquid nitrogen immediately. Several glass beads (2–3 mm) were added after the material was frozen. The mycelium was ground two times 10 s in a bead beater machine, in between the material was refrozen in liquid nitrogen and some new glass beads were added. The ground material was used in a standard phenol–chloroform extraction (Sambrook et al. 1989).

The *het-c* gene was amplified with standard PCR, using primers that bind roughly 500 bp before and after the transcribed sequence of *het-c*. Primers used were as follows: 3′-CGAAGGTGAAAACGAGACGA-5′ and 3′-ACCAAGGCTGGACCTGATA-5′. Based on the sequence of *het-c1* (GenBank: L36207), these primers are expected to give a product of 1,792 bp. PCR was done with following cycle conditions: 5 min at 95 °C followed by 35 cycles of 30 s at 94 °C, 30 s at 50 °C, and 2 min at 72 °C, followed by one cycle of 5 min at 72 °C and a final hold step at 4 °C. PCR products were cleaned using the Sigma GenElute PCR clean-Up Kit. Cleaned PCR products were sequenced using the value read service of MWG biotech. Sequencing was done in two directions using primers binding just outside the coding part of *het-c*. Primers used for sequencing were as follows: 3′-CAACCAACCTTCAACCAACC-5′ and 3′-CAGATGCGTATGCTTTTTGC-5′. These primers bind just outside the ORF of the *het-c* gene. Sequences were reliable up to 900 bp from the starting point. Sequence data were handled with the DNAstar software package. 

Sequences for *het-c5* to *het-c11* have been deposited in GenBank with accession numbers KF951052 until KF951058.

### Sequence Analyses

Alignments of the DNA and amino acid sequences were done with ClustalW (Larkin et al. 2007). Phylogenetic trees were constructed using the Neighbor-Joining method with the software package MEGA4 (Tamura et al. 2007). Bootstrap supports, expressed as a percentage, were calculated over 1,000 replicates. Additionally, the sequences were analyzed for possible positive diversifying selection on *het-c* codons. To detect selection, we used the methods developed by Yang et al. (2000), which are implemented in the HyPhy software (Kosakovsky Pond and Muse 2005). Besides testing the *het-c* alleles, a set of 37 GLTP encoding genes from filamentous fungi found by a Blast search of the Fungal Genome Database held at the NCBI, including one *P. anserina het-c* allele, was tested for positive diversifying selection. The sequence accession numbers are listed in supplementary figure S8, Supplementary Material online.

### Transformation and Testing Functionality of New *het-c* Alleles

Protoplasts were prepared and transformed as previously described (Bergès and Barreau 1989). The recipient *Δhet-c P. anserina* strain lacks the entire *het-c* ORF (Saupe et al. 1994). The PCR products made for sequencing of the new *het-c* variants were cloned in the pGEM-t easy vector (Promega). 7.5 μg of the cloned *het-c* DNA was used to cotransform protoplasts of the *Δhet-c* strain with 1.5 μg of the pPaBle plasmid conferring resistance to phleomycin (Coppin and Debuchy 2000). The transformed protoplasts were spread on synthetic medium with a high sucrose concentration and phleomycin for selection of transformants. After 4 to 5 days, transformants were transferred to a standard synthetic medium.

For each new *het-c* allele, 18 transformants were tested for barrage formation with tester strains each expressing a different *het-e* or *het-d* allele. For each allele, two cotransformants have been checked for the presence of the introduced *het-c* allele by PCR using primers binding on the pGEM-t easy vector.

### Analysis of WD-Repeat Number Variation for Escaped Sectors of Self-Incompatible Progeny

Crosses were set up between the tester strain for *het-d2* and two different transformants with the incompatible alleles *het-c9* and *10.* Self-incompatible progeny were selected and transferred to PASM with 6 g/l dihydrostreptomycin covered with a cellophane sheet, which allows growth of the self-incompatible colonies. The cellophane sheets were then transferred to PASM without dihydrostreptomycin after 1 day of growth. After several days, escaping sectors were observed from these colonies. DNA was isolated from mycelium of these sectors and PCR on the WD repeat region was performed as was described by Paoletti et al. (2007).

## Supplementary Material

Supplementary data S1 and figures S1–S8 are available at *Molecular Biology and Evolution* online (http://www.mbe.oxfordjournals.org/).

Supplementary Data
