# Supplementary material for: Natural Variation of Heterokaryon Incompatibility Gene het-c in Podospora anserina Reveals Diversifying Selection
Source: Mol Biol Evol. 2014 Jan 20;31(4):962–74. doi: 10.1093/molbev/msu047 (PMC3969566; doi:10.1093/molbev/msu047)
Supplement: Supplementary Data [file supp_31_4_962__index.html]

Natural variation of heterokaryon incompatibility gene het-c in Podospora anserina reveals diversifying selection — Natural Variation of Heterokaryon Incompatibility Gene het-c in Podospora anserina Reveals Diversifying Selection — Natural Variation of Heterokaryon Incompatibility Gene het-c in Podospora anserina Reveals Diversifying Selection — Supplementary Data 

# Natural Variation of Heterokaryon Incompatibility Gene *het-c* in *Podospora anserina* Reveals Diversifying Selection

## Supplementary Data

files

**Files in this Data Supplement:**

- Supplementary Data - pdf file
