# Supplementary material for: Natural Variation of Heterokaryon Incompatibility Gene het-c in Podospora anserina Reveals Diversifying Selection
Source: Mol Biol Evol. 2014 Jan 20;31(4):962–74. doi: 10.1093/molbev/msu047 (PMC3969566; doi:10.1093/molbev/msu047)
Supplement: Supplementary Data [file supp_msu047_supplemental_figures.pdf]

Supplemental figure 1

ClustalW alignment of the *het-c* alleles nucleic sequences. Sequences of the introns are indicated in italics. Only polymorphic sites are indicated compared to the *het-c1* reference sequences for the variant alleles. Synonymous mutations are given in bold.

het-c1 1 ATGGCCGCCGCCGCCGTTGTCCAAAT**C**CCCGTCGGGGCTACCTTCCTTAAGACCTTCAAGAAGTCCTTCGTT 72
het-c2 1 -------------------------------C----------------G----------------------- 72
het-c3 1 -- 72
het-c4 1 -------------------------------C-- 72
het-c5 1 -- 72
het-c6 1 -- 72
het-c7 1 -------------------------------C----------------G----------------------- 72
het-c8 1 -- 72
het-c9 1 -- 72
het-c10 1 -------------------------------C----------------G----------------------- 72
het-c11 1 --------------------------**T**----C----------------G----------------------- 72

het-c1 73 GATGTCCCAATCGATGC**T**GAGAAGGGCAATGCCATCTCCACCGCCGAGTTCCTTGAGGCGGCCGAGTCCTTG 144
het-c2 73 -- 144
het-c3 73 -- 144
het-c4 73 -----------------**C**-- 144
het-c5 73 -- 144
het-c6 73 -- 144
het-c7 73 -----------------**C**-- 144
het-c8 73 -- 144
het-c9 73 -- 144
het-c10 73 -----------------**C**-- 144
het-c11 73 -----------------**C**-- 144

het-c1 145 ACCACCATGTTCGATGTGCTCGGCTCCATCGCCTTCACCCC**C**GTCAAGACGGATATGTTGGGCAACGTCGAG 216
het-c2 145 ------------------------------------T----------------------------------- 216
het-c3 145 -- 216
het-c4 145 ------------------------------------T------------A---------------------- 216
het-c5 145 -- 216
het-c6 145 ------------------------------------T----------------------------------- 216
het-c7 145 ---------------------------G--------T------------A---------------------- 216
het-c8 145 -- 216
het-c9 145 ---TT--------- 216
het-c10 145 ------------------------------------T----**T**-------A---------------------- 216
het-c11 145 ---------------------------G--------T------------A---------------------- 216

het-c1 217 *GTGCGCTGCTATTATACCCGTTGGTTAGGACGTCAGCTAACCACCCTTTCCTGCTAG*AAAATTCGCAAGCGC 288
het-c2 217 ------------------*T*--- 288
het-c3 217 -- 288
het-c4 217 -- 288
het-c5 217 -- 288
het-c6 217 -- 288
het-c7 217 ---------------------------*T*--*T*-----------------------------------C----- 288
het-c8 217 -- 288
het-c9 217 -- 288
het-c10 217 -- 288
het-c11 217 -- 288

het-c1 289 ATGCTTGCCGCCCCCCTCGCATCCCAGAACATCCAGGATCTTGTGAGGAACGAGCTCAAGACCAAAAGCCAT 360
het-c2 289 -------------------AG--- 360
het-c3 289 -- 360
het-c4 289 -------------------A-- 360
het-c5 289 -- 360
het-c6 289 -- 360
het-c7 289 -------------------AG--- 360
het-c8 289 -- 360
het-c9 289 -- 360

het-c10 289 -------------------A-- 360
het-c11 289 -------------------AG--- 360

het-c1 361 ACCGCGACGGAGGGGTTGCTGTGGCTGGTCAG*GTGAGGGCGCCTTGATCTGTTCCATAACCAAGCCAAGTTA* 432
het-c2 361 --*G*-----*T*-----*C*- 432
het-c3 361 -- 432
het-c4 361 ---*CC*- 432
het-c5 361 -- 432
het-c6 361 -- 432
het-c7 361 ---*G*----*G*-----*T*------- 432
het-c8 361 -- 432
het-c9 361 -- 432
het-c10 361 --*G*-----*T*------- 432
het-c11 361 --*G*-----*T*---*A*-*C*- 432

het-c1 433 *CTGACAGTGGTCTGTGACAG*GGGTCTCGAATTCACATGCATTGCTCTTAGCAAGAACATCAACTCAACAGAG 504
het-c2 433 --GG---------- 504
het-c3 433 -- 504
het-c4 433 --G----------- 504
het-c5 433 -- 504
het-c6 433 -- 504
het-c7 433 ---G-T------GG-------A-- 504
het-c8 433 ------------------------------------C----------------------------------- 504
het-c9 433 -- 504
het-c10 433 --T-------GG---------- 504
het-c11 433 --GG---------- 504

het-c1 505 GAGCTCGCCGACTCCTTCCGCGGGTCTTACAGTGAGACTCTCATGCGACACCACAGCTTCCTGGTGAAGCGC 576
het-c2 505 --------------------------------G-T--------A--C-----------------------C- 576
het-c3 505 ---------C-- 576
het-c4 505 --C- 576
het-c5 505 -----------G-- 576
het-c6 505 --C- 576
het-c7 505 -------A--C----------AA-----------T--------A--C-----------------------C- 576
het-c8 505 -- 576
het-c9 505 ---------A--C- 576
het-c10 505 ------------------G---------------C-----------------------------------C- 576
het-c11 505 ----------------------------------T-----------------------------------C- 576

het-c1 577 ATCTTCAGCGCCGCCATGGGCGCGTGCCCATACCGCAAGGACTTCTACGCCAAGCTTGGTGACGA**C**GAGCAA 648
het-c2 577 ------------------A--- 648
het-c3 577 -- 648
het-c4 577 -- 648
het-c5 577 -- 648
het-c6 577 -- 648
het-c7 577 ------------------A--**T**------ 648
het-c8 577 -- 648
het-c9 577 -- 648
het-c10 577 ------------------A-A--- 648
het-c11 577 ------------------A--- 648

het-c1 649 AAGGTTCAAGAGGAGCTTCGCGAATACCTTGCTGCTCT**C**GACAAGAT**C**GTCTACATTCTCAAGGGATTCTTG 720
het-c2 649 -------------------------------T-------------------A-----------A-------- 720
het-c3 649 -- 720
het-c4 649 -- 720
het-c5 649 -- 720
het-c6 649 -- 720
het-c7 649 --------------------------------------**T**--------**T**---A-----------C-------- 720
het-c8 649 -- 720
het-c9 649 -- 720
het-c10 649 ---------------------A-----------------------------A-------------------- 720
het-c11 649 ---A-------------------- 720

```
het-c1      721 GAGAGCAAGGAGGCCAAGTGGTAA  744
het-c2      721 ------------------------  744
het-c3      721 ------------------------  744
het-c4      721 ------------------------  744
het-c5      721 ------------------------  744
het-c6      721 ------------------------  744
het-c7      721 ------------------------  744
het-c8      721 ------------------------  744
het-c9      721 ------------------------  744
het-c10     721 ------------------------  744
het-c11     721 ------------------------  744
```

Supplemental figure 2:

Incompatibility patterns of the eleven known naturally occurring *het-c* alleles (data for *het-c1* to *het-c4* taken from (Bernet 1967) and of eleven chimeric alleles artificially constructed from *het-c1* to *het-c4* (taken from (Saupe, Turcq, Bégueret 1995)). Interactions were tested with the four available *het-e* and three *het-d* alleles. A dark grey square indicates an incompatible reaction was shown for this combination of alleles. If an allele has a similar incompatibility pattern as an allele earlier in the list, it is indicated by the "=" symbol followed by the allele number it shows similarity to. Alleles with the same incompatibility pattern are given the same background color.

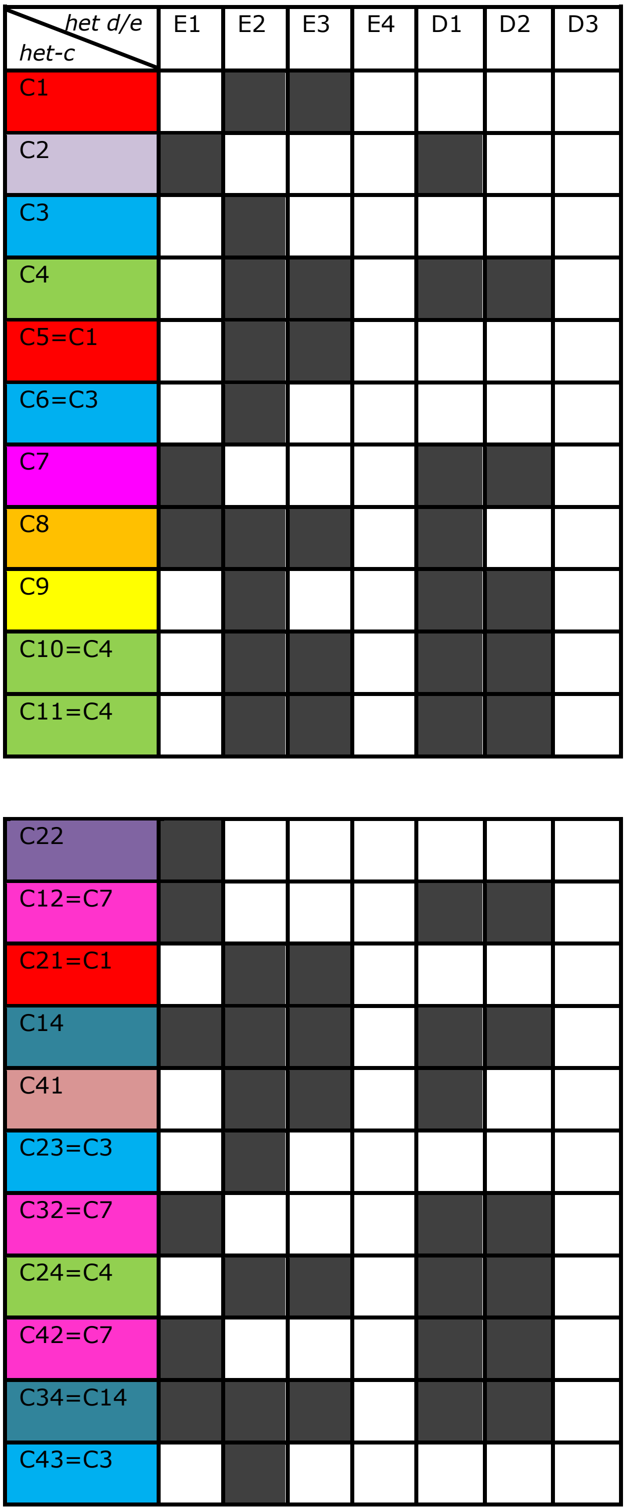

Supplemental figure 3

Distribution of *het-c*, *-d* and *-e* alleles in the *P. anserina* isolates collected in France in the 1940s. A\ incompatibility patterns of the four *het-c* alleles found in these isolates. B\ - D\ pie charts showing the distribution of alleles for respectively *het-c*, *het-d* and *het-e*. Numbers represent amount of isolates with this allele.

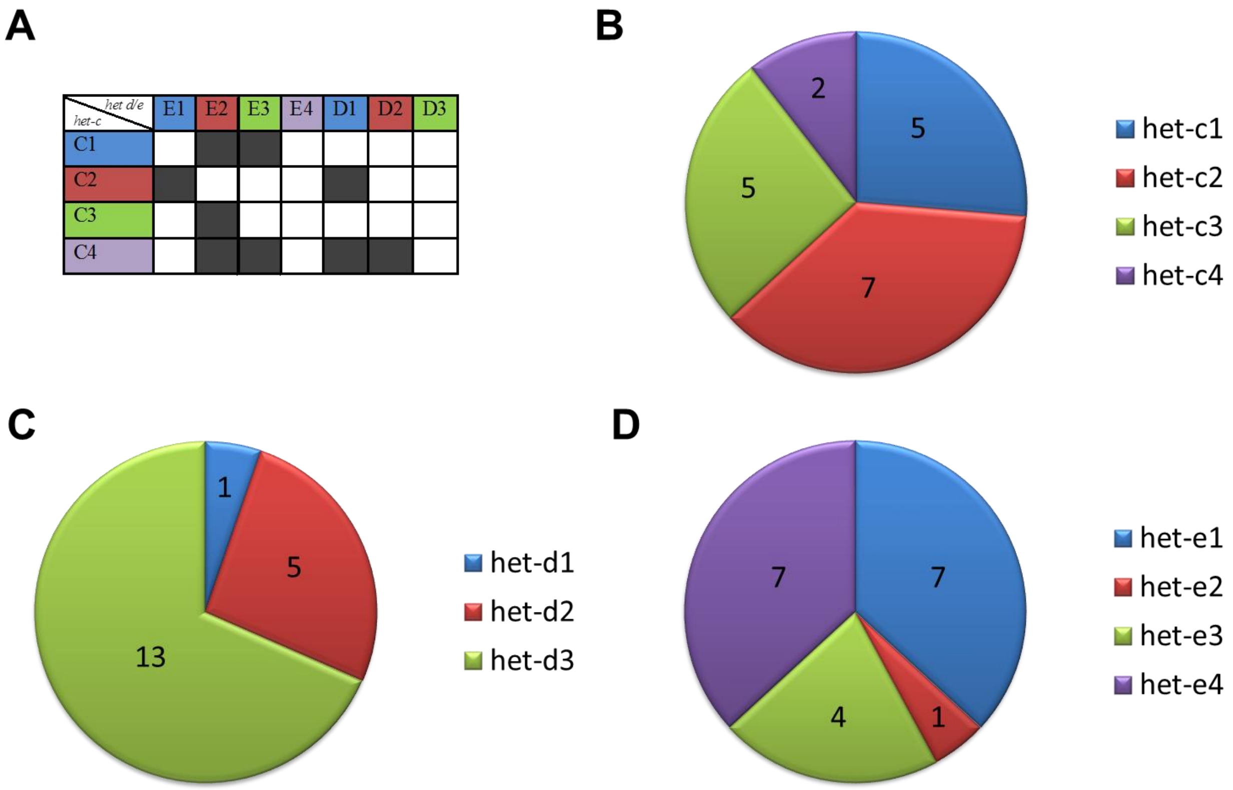

Supplemental figure 4:

Distribution of the pAL2-1 plasmid in relation with the *het-c* alleles, *het-c* phenotypic classes (C1, C2 or C3) or incompatibility with the *het-e1* and *het-e2* testers. We combined our data on *het-c* allele distribution in the Wageningen *P. anserina* population with the distribution of the pAL2-1 senescence plasmid (van Diepeningen et al. 2008) resulting in a total of 95 isolates characterised for both sets of data. The proportion of infected and non-infected isolates is represented depending on A/ the *het-c* alleles expressed, B/ the C-type to which the *het-c* alleles belong, C/ the incompatibility with *het-e1* or *het-e2*. In the latest panel the difference between infected and non-infected isolates is not significant (2 tail Fisher's exact test, p value=0.145).

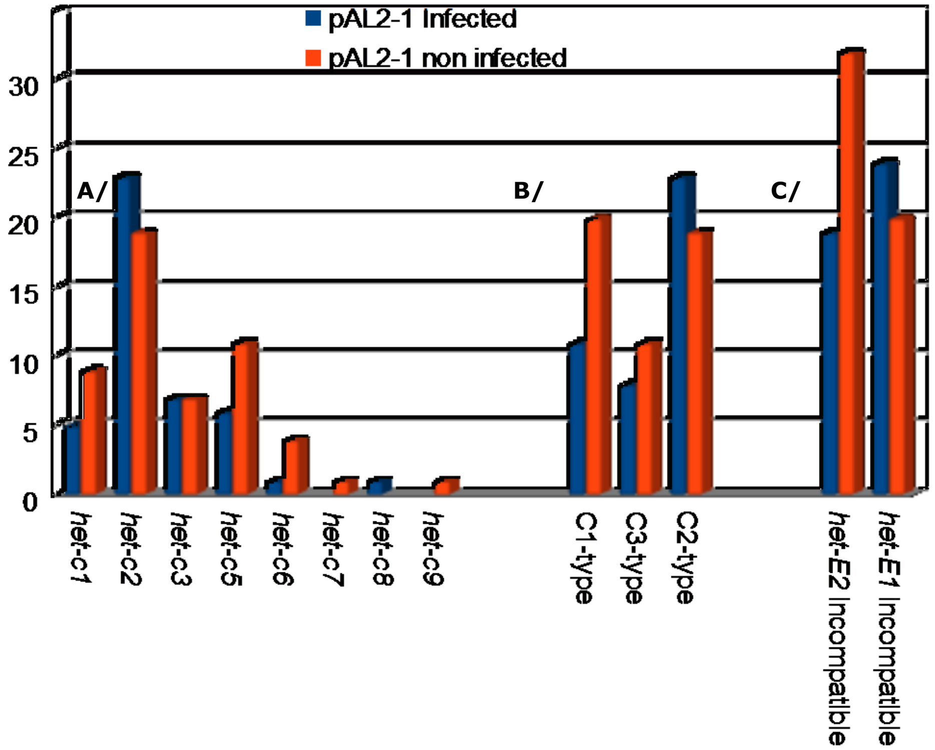

Supplemental figure 5:

A/ Accession numbers for the GLTP encoding cDNAs from filamentous fungi used. B/ Protein guided Neighbor-joining phylogenetic tree constructed from the GLTPs encoding cDNAs.

A/ Podospora anserina het-c2 (U05236), *Chaetomium globosum* CBS148.51 (XM_001224167), *Glomerella graminicola* M1 (GG697350), *Verticillium albo_atrum* VaMs (XM_003001318), *Metarhizium acridum* CQMa102 (GL698531), *Neurospora crassa* OR74A (XM_956987), *Sclerotinia sclerotiorum* 1980 UF_70 (XM_001585194), *Nectria haematococca* mp VI77_13_4 (XM_003040028), *Phaeosphaeria nodorum* SN15 (XM_001796420), *Fusarium oxysporum* Fo5176 (AFQF01002826), *Arthroderma gypseum* CBS118893 (XM_003176546), *Trichophyton verrucosum* HKI0517 (XM_003022135), *Trichophyton rubrum* CBS118892 (XM_003238513), *Trichophyton equinum* CBS 127.97 (DS995722), *Pyrenophora tritici_repentis* Pt_1C_BFP (XM_001935155), *Trichophyton tonsurans* CBS112818 (GG698505), *Pyrenophora teres f. teres* 0_1 (XM_003303860), *Arthroderma benhamiae* CBS112371 (XM_003016258), *Aspergillus terreus* NIH2624 (XM_001213225), *Penicillium chrysogenum* Wisconsin 54_1255 (XM_002559878), *Aspergillus niger* CBS51388 (XM_001400048), *Aspergillus clavatus* NRRL1 (XM_001271363), *Neosartorya fischeri* NRRL181 (XM_001263079), *Arthroderma otae* CBS113480 (XM_002849967), *Coccidioides posadasii* C735deltaSOWgp (XM_003066772), *Aspergillus fumigatus* Af293 (XM_749149), *Coccidioides immitis* RS (XM_001246321), *Paracoccidioides brasiliensis* Pb01 (XM_002791993), *Aspergillus nidulans* FGSCA4 (XM_655659), *Ajellomyces capsulatus* NAm1 (XM_001544035), *Uncinocarpus reesii* 1704 (XM_002540637), *Ajellomyces dermatitidis* SLH14081 (XM_002623113), *Aspergillus flavus* NRRL 3357 (AFL2G_03641), *Aspergillus oryzae* RIB40 (AO090012000776), *Fusarium graminearum* PH-1 (FG01974), *Magnaporthe grisea* (XM_364530).

B/ Neighgour joining phylogenetic tree of the fungal GLTPs listed above. The sequences were aligned with ClustalW and the tree constructed with the package MEGA5.

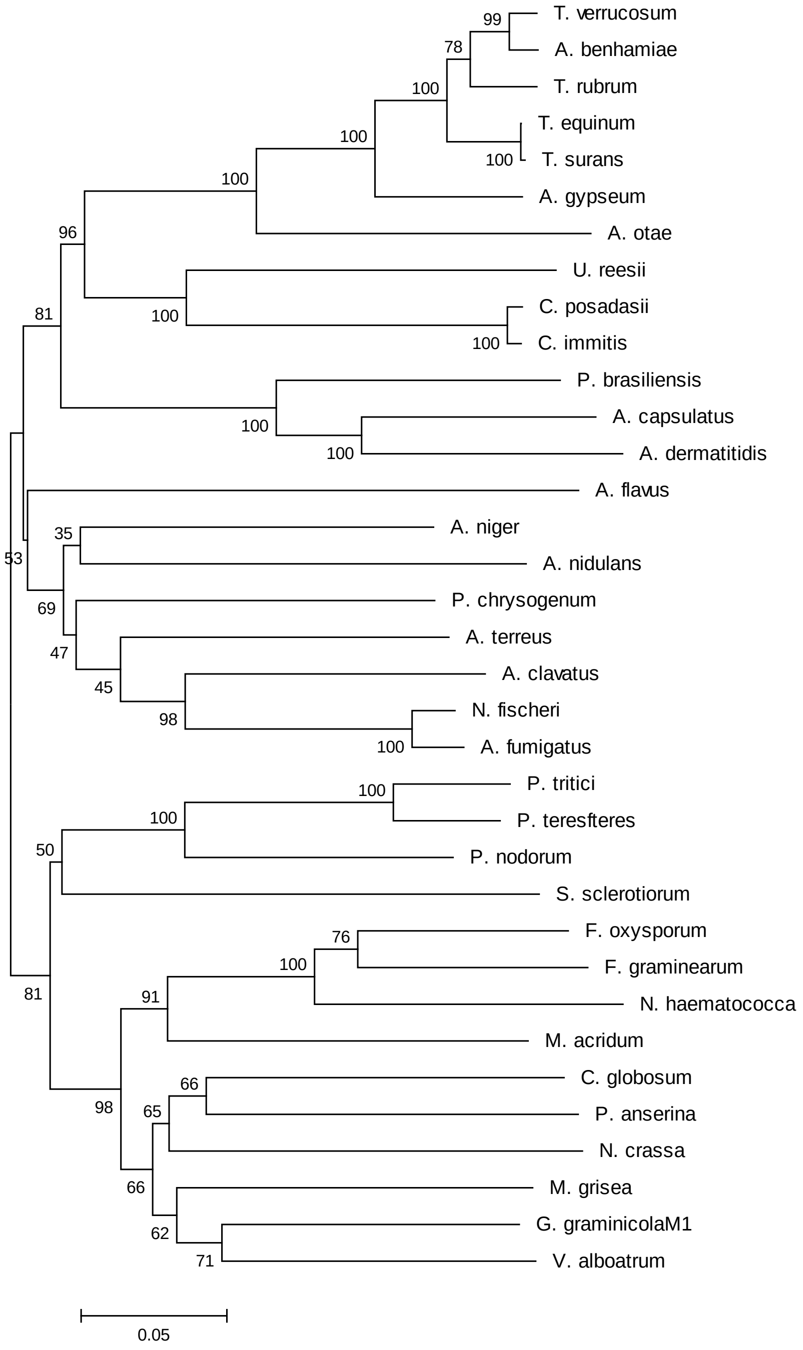

Supplemental figure 6

Polymorphic sites identified in alleles of *N. crassa* NCU07947 orthologous to the *P. anserina het-c* gene. NCU07947 is located on the minus strand of supercontig 4 at coordinates 1260122-1260907. Polymorphisms are highlighted and represented on the reference sequence from *N. crassa* isolate OR74. Positions are relative to start of the ORF. Data are extracted from Ellison *et al.* (Ellison et al. 2011).

```
atgtctaccggccagatcccccccggaggtacctacctcaacaccctcaagcgttccttc
 M  S  T  G  Q  I  P  P  G  G  T  Y  L  N  T  L  K  R  S  F

                                   T96               t114
accgacgtccccgtccaggccgacaatgggaatgccattcccaccaccgagttcctcgag
 T  D  V  P  V  Q  A  D  N  G  N  A  I  P  T  T  E  F  L  E

                          T147                    t171
gctgccgagtcgctagtctccatcttcgatgtgctcggctctgccgccttctcccccgtc
 A  A  E  S  L  V  S  I  F  D  V  L  G  S  A  A  F  S  P  V

                                   T216     t225        t237
aagagcgacatgctcggcaatgtcgagaaaatccgccagcgtttccttgccgccccgacc
 K  S  D  M  L  G  N  V  E  K  I  R  Q  R  F  L  A  A  P  T

                                      G279  t285
gagtccgagactctccaggaccttgtgaacaacgagcaaaaggccaaggagaacaaggcc
 E  S  E  T  L  Q  D  L  V  N  N  E  Q  K  A  K  E  N  K  A

                                                  C351
ggccaggccctgctctggctcgtcaggggtctcgagttcacttgcaaaggtcttgccaac
 G  Q  A  L  L  W  L  V  R  G  L  E  F  T  C  K  G  L  A  N

                             c390  c396        t408
aacgtcgccgccgctgaccaggagctttctacctcgttccgcgctgcctacgatgtcacc
 N  V  A  A  A  D  Q  E  L  S  T  S  F  R  A  A  Y  D  V  T

        T429           t444  a450
ctcaagcccaccacagcttcctcatcaagcccatcttcagcgccgccatgagcgcttgc
 L  K  P  H  H  S  F  L  I  K  P  I  F  S  A  A  M  S  A  C

                    C501                                c537
ccttaccgcaaggacttctataccaagctcggtgacgaccaggacaaggtcaacgctcag
 P  Y  R  K  D  F  Y  T  K  L  G  D  D  Q  D  K  V  N  A  Q

ctcaaggagtacctcgcagctctcgagaactttgtcaacatcctcaaggccttcctcgac
 L  K  E  Y  L  A  A  L  E  N  F  V  N  I  L  K  A  F  L  D

agcaagggcatcaagaaatag
 S  K  G  I  K  K  -
```

Supplemental figure 7

Polymorphic sites identified in alleles of the locus *Coccidioides* species reported to the cDNA sequence of the locus CPSG_04807 from the *C. posadasii* RMSCC757/Silveira (access number EFW18121) orthologous to the *P. anserina het-c* gene. Synonymous polymorphisms are highlighted in pink and a single non synonymous polymorphism is highlighted in green and represented on the reference sequence from *N. crassa* isolate OR74. Positions are relative to the start of the gene. Two polymorphic sites not represented here are located in intronic sequences. Data are extracted from Ellison *et al.* (Ellison et al. 2011).

```
atggcttctgctgctgtgattcccgccgacggcacctggttcgacactatcaggaggtca
 M  A  S  A  A  V  I  P  A  D  G  T  W  F  D  T  I  R  R  S

ttcgccgatgtcccaatcaacgacaacggtatttccacaacggagttcctcgaggctgcc
 F  A  D  V  P  I  N  D  N  G  I  S  T  T  E  F  L  E  A  A
                 g138               g205
gaagctttggtgatgctatttgacctccttggctccgtcgcattcactccggtaaagaac
 E  A  L  V  M  L  F  D  L  L  G  S  V  A  F  T  P  V  K  N

gacttgctaggcaacatcaagaaaattcgtgaccgccagttggcagctccagcagaatcc
 D  L  L  G  N  I  K  K  I  R  D  R  Q  L  A  A  P  A  E  S
                             a376                       c403
gagactcttcagcaacttgttgtgaacgagctcaagactggaaagcacacggcgactgaa
 E  T  L  Q  Q  L  V  V  N  E  L  K  T  G  K  H  T  A  T  E

ggcctgctatggctggtccgtggtctcgacttcactgcccaagccctccgcctcaatctt
 G  L  L  W  L  V  R  G  L  D  F  T  A  Q  A  L  R  L  N  L
                    c553
tccgatcctgctgctgaactttctacttctttccgtgctgcatacggcactaccttaaaa
 S  D  P  A  A  E  L  S  T  S  F  R  A  A  Y  G  T  T  L  K
           t604  g610
ccacatcacggccttgcttgtcaaaccgattttcagtgctgccatgtccgcaactccttat
 P  H  H  G  L  L  V  K  P  I  F  S  A  A  M  S  A  T  P  Y

agaaaggacttctatgccaagcttggtcaggacgctaccaaggtctcaactgcgatgaac
 R  K  D  F  Y  A  K  L  G  Q  D  A  T  K  V  S  T  A  M  N
g713           c727
atcgaaataactgctttggagaaagtagtaggaatcctccatgaattccttaaaagtcca
 I  E  I  T  A  L  E  K  V  V  G  I  L  H  E  F  L  K  S  P
 V

gcagccaaatggtga
 A  A  K  W
```

Supplemental figure 8

Different fungal species were checked for polymorphisms in their *het-c* homologues in different isolates. The alleles were identified by tblastn searches of the NCBI database using *P. anserina* HET-C2 as query. Positively selected sites predicted by model M2 are highlighted in red, additional sites predicted by model M8 are highlighted in grey.

A/ *Paracoccidioides brasiliensis :* ClustalW alignment of HET-C2 protein and variant homologues from *P. brasiliensis*. Polymorphic sites between variants *P. brasiliensis het-c* homologues are indicated in yellow, while sites under diversifying selection are highlighted in grey. Accession numbers are indicated in brackets.

P. brasiliensis PB01 (XP_002792039)
P. brasiliensis PB03 (EEH23613)

```
PB01                          --MASTQVIPQGGTWFDTLNRGFYSVRIADD--NAISTTEFLEAAESLTT 46
PB03                          --MASTQVIPQGGTWFDTLNRGFYNVRITDD--NAISTTEFLEAAESLTT 46
HET-C                         MAAAAVVQIPAGATFLETFKKSFVDVPIDAEKGNAISTAEFLEAAESLTT 50
                                 *:.  ** *.*:::*:::.* .* *  :  *****:***********

PB01                          LFDLLGSVAFTPVKNDLLGNIKKIRDRQLAAPAESETLQALVLNELKAKK 96
PB03                          LFDLLGSVAFTPVKNDLLGNIKKIRDRQLAAPAESETLQELVLNELKAKK 96
HET-C                         MFDVLGSIAFSPVKTDMLGNVEKIRKRMLAAPLESQNIQDLVRNELKTKS 100
                              :**:***:**:***.*:***::***.* **** **:.:* ** ****:*.

PB01 PB01                     NTATVGLLWLVRGLDFTAQALRHNISNPTDELSASFRVAYGKTLKPYHNF 146
PB03                          NTATVGLLWLVRGLDFTAQALRHNISNPADELSASFRVAYGKTLKPYHNF 146
HET-C                         HTATEGLLWLVRGLEFTCIALSKNIG-STEELADSFRGSYRVTLKPHHSF 149
                              :*** *********:**. ** :**. .::**: *** :*  ****:*.*

PB01                          LIKPIFTAAMGATPYRKDFYANLGDDSVKSQAALELSTTSLEKIVSILKE 196
PB03                          LIKPIFTAAMGATPYRKDFYAKLGDDSVKSQAALELSTTSLEKIVSILKE 196
HET-C                         LVKPIFSAAMSACPYRKDFYAKLGDDEQKVQEELREYLVALDKIVNILKR 199
                              *:****:***.* ********:****. * *  *.   .:*:***.***.

PB01                          FLETPEVKKAAS 208
PB03                          FLETPEVKKAAS 208
HET-C                         FLESKEAKW--- 208
                              ***: *.*
```

B/ Ajellomyces capsulatus : ClustalW alignment of HET-C2 protein and variant homologues from *A. capsulatus*. Polymorphic sites between variants *A. capsulatus het-c* homologues are indicated in yellow, while sites under diversifying selection are highlighted in grey. Accession numbers are indicated in brackets.

A. capsulatus Nam1 (XP_001544085)
A. capsulatus H88 (EGC47776)
A. capsulatus H143 (EER38624)

```
Nam1                          --MANTQVIPEGGTWFDTLKRGFDAVPVADD--NAISTSEFLEAAESLTT 46
H88                           --MANTQVIPEGGTWFDTLTRGFDAVPVADD--NAISTSEFLEAAESLTT 46
H143                          --MANTQVIPEGGTWFDTLTRGFDAVPVADD--NAISTSEFLEAAESLTT 46
HET-C                         MAAAAVVQIPAGATFLETFKKSFVDVPIDAEKGNAISTAEFLEAAESLTT 50
                                 * .  ** *.*:::*:.:.*  **:  :  *****:***********

Nam1                          LF-----VAFTPVKNDLLGNIKKIRDRQLAAPAESETLQALVLNEIKAKK 91
H88                           LFDLLGSVAFTPVKNDLLGNIKTACG-----TAESETLQALVLNEIKAKK 91
H143                          LFDLLGSVAFTPVKNDLLGNIKKIRDRQLAAPAESETLQALVLNEIKAKK 96
HET-C                         MFDVLGSIAFSPVKTDMLGNVEKIRKRMLAAPLESQNIQDLVRNELKTKS 100
                              :*     :**:***.*:***::.        . **:.:* ** **:*:*.

Nam1                          NTASVGFLWLVRGLDFTAKALRHNISFPNDELSTSFRAAYGDTLKPHHNF 141
H88                           NTASVGFLWLVRGLDFTAKALRHNISFPNDELSTSFRAAYGDTLKPHHNF 141
H143                          NTASVGFLWLVRGLDFTAKALRHNISFPNDELSTSFRSSLR--------- 137
HET-C                         HTATEGLLWLVRGLEFTCIALSKNIG-STEELADSFRGSYRVTLKPHHSF 149
                              :**: *:*******:**. ** :**. ..:**: ***.:

Nam1                          LVKPIFVAAMGATPYRKDFYAKLGNDTAKCQAALELSTASLEKIVSILKE 191
H88                           LVKPIFVAAMGATPYRKDFYAKLGNDPAKCQAALELSTASLEKIVSILKE 191
H143                          --------------------------------------------------
HET-C                         LVKPIFSAAMSACPYRKDFYAKLGDDEQKVQEELREYLVALDKIVNILKR 199


Nam1                          FLETPEVKKACS 203
H88                           FLETPEVKKACS 203
H143                          ------------
HET-C                         FLESKEAKW--- 208
```

C/ Ajellomyces dermatidis: ClustalW alignment of HET-C2 protein and variant homologues from *A. dermatidis*. Polymorphic sites between variants *A. dermatidiis het-c* homologues are indicated in yellow, while sites under diversifying selection are highlighted in grey. Accession numbers are indicated in brackets.

A. dermatidis 18188 (EGE78761)
A. dermatidis SLH14081 (XP_002623159)

```
18188             --MANTQVIPPGGTWFDTLQRGFDAVPVSDD--NAITTSEFLEAAEALTTLFDLLGSVAF 56
SLH14081          --MANTQVIPPGGTWFDTLQRGFDAVPVSDD--NAITTSEFLEAAEALTTLFAFTGANYT 56
HET-C             MAAAAVVQIPAGATFLETFKKSFVDVPIDAEKGNAISTAEFLEAAESLTTMFDVLGSIAF 60
                     * .  **.*.*:::*:::.*  **:. :  ***:*:*******:***:* . *:

18188             TPVKNDLLGNIKKIRDRQLAAPAESETLQALVLNELKAKKNTASGGLLWLLRGLDFTAKA 116
SLH14081          IPFQ--------KIRDRQLAAPAESETLQALVLNELKAKKNTASGGLLWLLRGLDFTAKA 108
HET-C             SPVKTDMLGNVEKIRKRMLAAPLESQNIQDLVRNELKTKSHTATEGLLWLVRGLEFTCIA 120
                   *.:        ***.* **** **:.:* ** ****:*.:**: *****:***:**. *

18188             LRHNISHPNEELSASFRAAYGDTLKPHHNFLVKPIFVAAMGATPYRKDFYRKLGDDPAKC 176
SLH14081          LRHNISHPNEELSASFRAAYGDTLKPHHNFLVKPIFVAAMGATPYRKDFYRKLGDDPAKC 168
HET-C             LSKNIGS-TEELADSFRGSYRVTLKPHHSFLVKPIFSAAMSACPYRKDFYAKLGDDEQKV 179
                  * :**.  .***: ***.:*  ******.******* ***.* ******* *****  *

18188             QAALELSTASLEKIVSILKDFLETPEVKKAIS 208
SLH14081          QAALELSTASLEKIVSILKDFLETPEVKKAIS 200
HET-C             QEELREYLVALDKIVNILKRFLESKEAKW--- 208
                  *  *.   .:*:***.*** ***: *.*
```

D/ *Aspergillus fumigatus*. ClustalW alignment of HET-C2 protein with homologues identified in *A. fumigatus* Af293 and Af1163 strains. No polymorphic sites were identified between these two allele expressed in the sequenced strains A1163 (AFUB_035370) and strain AF293 (Afu3g13820).

A. fumigatus A1163 (AFUB_035370)
A. fumigatus AF293 (Afu3g13820)

```
Af293           MAAA----IPPGGTWFDTLKRSFADVPIND---NGISTTEFLEAAESLVTLFDLLGSKCF 53
Af1163          MAAA----IPPGGTWFDTLKRSFADVPIND---NGISTTEFLEAAESLVTLFDLLGSKCF 53
het-c           MAAAAVVQIPAGATFLETFKKSFVDVPIDAEKGNAISTAEFLEAAESLTTMFDVLGSIAF 60
                ****    **.*.*:::*:*:**.****:    *.***:*********.*:**:*** .*

Af293           APVKNDLLGNIKKVRDRQLAAPAESETLQALVVNELKTGKHVATEGLLWLVRGLDFTVQA 113
Af1163          APVKNDLLGNIKKVRDRQLAAPAESETLQALVVNELKTGKHVATEGLLWLVRGLDFTVQA 113
het-c           SPVKTDMLGNVEKIRKRMLAAPLESQNIQDLVRNELKTKSHTATEGLLWLVRGLEFTCIA 120
                :***.*:***::*:*.* **** **:.:* ** ***** .*.************:**  *

Af293           LRHNLDKETELSVSFREAYGNTLKPHHSFVVKPIFSAAMSATPYRKEFYEKLGSDSDKVN 173
Af1163          LRHNLDKETELSVSFREAYGNTLKPHHSFVVKPIFSAAMSATPYRKEFYEKLGSDSDKVN 173
het-c           LSKNIGSTEELADSFRGSYRVTLKPHHSFLVKPIFSAAMSACPYRKDFYAKLGDDEQKVQ 180
                * :*:..  **: *** :*  ********:*********** ****:** ***.*.:**:

Af293           VALKREVEALEKIVATLNAFMSSKEAKW 201
Af1163          VALKREVEALEKIVATLNAFMSSKEAKW 201
het-c           EELREYLVALDKIVNILKRFLESKEAKW 208
                  *:. : **:***  *: *:.******
```
